# The epidemiology and burden of respiratory syncytial virus and influenza infections in hospitalized children under 5 years old in Zhejiang, China (2018–2023)

**DOI:** 10.3389/fpubh.2025.1470770

**Published:** 2025-03-19

**Authors:** Wanwan Sun, Qiuyao Duan, Lei Guo, An Zhu, An Tang, Ziping Miao, Yu Zhang, Fen Yuan, Xiaofei Fu, Suyan Shi, Lan Wang, Shijian Li, XiaoZhi Xu, Chunlei Zhu, Lefei Zhou, Li Rui, Pang Yue, Zhao Yu, Jinren Pan, Chaorong Ni, Shelan Liu

**Affiliations:** ^1^Zhejiang Key Lab of Vaccine, Department of Infectious Diseases, Infectious Disease Prevention and Control, Zhejiang Provincial Center for Disease Control and Prevention, Hangzhou, China; ^2^School of Public Health, Xiamen University, Xiamen, Fujian, China; ^3^Department of Infection Control, The Second Affiliated Hospital and Yuying Children’s Hospital of Wenzhou Medical University, Wenzhou, China; ^4^Department of Pediatrics, Second People’s Hospital of Jinyun County (Huzhen Branch of Lishui Central Hospital), Lishui, China; ^5^Department of Infectious Diseases, Zhoushan Center for Disease Control and Prevention, Zhoushan, China; ^6^Tonglu Hospital of the First People’s Hospital of Hangzhou, Hangzhou, China; ^7^Tonglu Maternal and Child Health Center, Hangzhou, China; ^8^Department of Infectious Diseases, Jiaxing Center for Disease Control and Prevention, Jiaxing, China; ^9^The Second People’s Hospital of Tonglu County, Hangzhou, China; ^10^Department of Geriatrics, First Affiliated Hospital, Zhejiang University School of Medicine, Hangzhou, China; ^11^Department of Public Health, The State University of New York at Old Westbury, New York, NY, United States; ^12^Department of Infectious Diseases, Wenzhou Center for Disease Control and Prevention, Wenzhou, China

**Keywords:** respiratory syncytial virus, influenza virus, pneumonia, disease burden, children

## Abstract

**Objective:**

Estimate changes in hospital-based respiratory syncytial virus (RSV) disease burden before and after the COVID-19 pandemic and compare this burden with influenza among children younger than 5 years old in China’s Zhejiang Province.

**Methods:**

We enrolled hospitalized children under 5 years old from eight hospitals in Zhejiang Province. Multiple testing methods were used to compare epidemiological characteristics, and multivariate logistic regression analyses were used to estimate the odds ratio (OR) and 95% confidence interval (CI) between the two groups.

**Results:**

In this study, of the 7,857 RSV and 2,571 influenza patients, the median age of the children was significantly lower for RSV infection than for influenza. Inpatients with RSV had longer hospitalization durations (mean: 5.66 days vs. 5.04 days; *p* < 0.001) and hospitalization costs (mean: 5,616.12 RMB vs. 5,352.99 RMB; *p* = 0.023) than those with influenza. RSV inpatients increased from 1,081 before the COVID-19 pandemic to 6,776 after the pandemic (*p* < 0.001), with 526.8% more hospitalizations than before the pandemic (*p* < 0.001). During 2020–2023, hospitalized children were older (16.86 months vs. 10.09 months; *p* < 0.001) and had a higher proportion of pneumonia (82% vs. 75% of hospitalized patients; *p* < 0.001) than during pre-pandemic seasons for children admitted due to RSV infection. However, the average RSV hospitalization cost was much lower after the pandemic (4,299.29 RMB vs. 5,697.51 RMB; *p* < 0.001). Compared with the prepandemic years (2018–2019), the influenza groups showed a similar trend; the number of inpatients increased during the 2020–2023 season (1,949 vs. 622, *p* < 0.001), with older ages (33.13 months vs. 27.42 months, *p* < 0.001), a lower proportion of pneumonia (38% vs. 45%, *p* < 0.001), and lower costs (3,631.03 RMB vs. 3,742.59 RMB, *p* < 0.001). RSV infection was related to a higher risk of hospitalization in all age groups, and the greatest risk was observed in the 6–12 month age group (OR = 23.1; 95% CI, 18.0–29.6), followed by the 5 months and younger group (OR = 22.4; 95% CI, 17.3–28.9), compared with influenza infection.

**Conclusion:**

RSV is a significant contributor to disease burden in hospitalized children under 5 years old, outweighing influenza. The COVID-19 pandemic impacted the epidemiological characteristics and disease burden of hospitalization for RSV and influenza infections. A more effective prevention strategy for both infections in young children, especially vaccinations against RSV and influenza, is urged.

## 1 Introduction

Respiratory syncytial virus (RSV) is an enveloped, single-stranded, negative RNA (Ribonucleic Acid) virus of the Pneumoviridae family of viruses (1). Globally, RSV is the most common pathogen responsible for lower respiratory tract infections (LRTIs) in children under 5 years old. It caused 33 million LRTI cases, 3.2–3.6 million hospitalizations, and more than 100,000 deaths annually before the coronavirus disease 2019 (COVID-19) pandemic (2). Notably, 99% of RSV deaths occur in low- and middle-income countries. China is one of the countries with a high prevalence of RSV, contributing nearly 50% of the global disease burden (2).

Treatment of RSV infection and measures to prevent infection should focus on children under 5 years because they are most vulnerable to RSV. In developed countries, either maternal RSV vaccination or infant immunization with RSV monoclonal antibodies prevents severe RSV disease in infants. In China, the long-acting monoclonal antibody Nirsevimab was officially approved by the National Medical Products Administration at the end of 2023 (3).

Globally, seasonal influenza circulates annually and causes substantial morbidity and mortality, with the highest burden among adults aged 65 years and older and children aged 5 years and younger (4). Every year, influenza causes an estimated 3–5 million cases of severe illness and 290,000–650,000 respiratory deaths. For children aged 5 years and younger, influenza is associated with 610,000–1,237,000 respiratory hospitalizations in the world annually (5). Influenza-associated infection (384 per 100,000 persons) or SARI (Severe Acute Respiratory Infection) (442–715 per 100,000 persons) hospitalization rate among children aged 5 years and younger is higher in China than in Singapore (186.8 per 100,000 persons) and Portugal (42.6 per 100,000 persons) (5). Although influenza vaccination can reduce the risk of illness caused by influenza virus infection by 40–60% among the general population, influenza vaccination is not included in China’s National Immunization Program, and vaccination coverage in the Chinese population is only approximately 2% (6).

The COVID-19 pandemic has drastically perturbed the epidemiology of RSV and influenza infections (7). In the first year of the COVID-19 pandemic, when nonpharmacological interventions (NPIs) were aggressively implemented, RSV and influenza cases immediately decreased worldwide (8). With NPI measures gradually relaxed, many countries, such as the USA and China, experienced an off-seasonal resurgence of RSV and influenza (9). Moreover, the testing model for respiratory infectious diseases in China was significantly changed from single to multipathogen by reverse transcription-polymerase chain reaction (RT-PCR) (10). All these public health measures may also have changed the disease characteristics and disease burden of other respiratory viruses, including RSV and influenza viruses.

China introduced a series of policies to control the influenza virus. First, the Chinese Center for Disease Control and Prevention recommends a yearly flu vaccine as the first and important action in reducing the risk of flu and its potentially serious outcomes. In China, the influenza vaccine was listed as the second-category vaccine that is self-paid and an optional inoculation; the coverage rate remained at a low level among children. Second, antiviral treatment may be considered in hospitals for symptomatic children with suspected or confirmed influenza disease, which has been covered by national medicare. For RSV, there were no RSV vaccines licensed in China, but the RSV antibody (Nirsevimab) was approved at the end of 2023 and has not been administrated largely to babies younger than 12 months of age. The etiology and epidemic characteristics of RSV were systematically monitored via a respiratory multipathogen monitoring system. Taking nonpharmaceutical interventions was most commonly used method for reducing the spread of influenza and RSV viruses in China.

To guide disease prevention strategies, it is critical to thoroughly characterize the evolving epidemiological patterns of RSV and influenza and the disease burden caused by the infection of the two viruses (11). Studies have explored RSV-related disease incidence, hospitalization rate, in-hospital mortality, and mortality rates (2, 9, 12, 13). Li Z-J (14) and Rha B (15) focused on analyzing the etiological composition of patients with respiratory tract infection, including acute respiratory infection (ARI) and acute lower respiratory tract infection, at different ages (including RSV and other respiratory viruses) and obtained the proportion of RSV in patients with respiratory tract infection. Studies by Shi T (16) and Sanz-Muñoz I (17) analyzed respiratory virus infection and the occurrence of pneumonia before and after COVID-19 nondrug intervention measures. Other studies analyzed the risk factors of severe RSV patients (18).

In summary, large-scale intensive comparative research on RSV and influenza infections in epidemiological characteristics and disease burden is still lacking in mainland China. In this present study, we collected data on over 7,000 RSV- and 2,000 influenza-infected hospitalized children aged 5 years and younger from multiple health care centers in China’s Zhejiang Province. we estimated the disease burden of influenza- and RSV-associated hospitalizations in prepandemic years vs. pandemic years and stratified the data by age, gender, underlying disease, and clinical severity.

## 2 Methods

### 2.1 Hospital selection

This multicenter retrospective cohort study was conducted between January 1, 2018 and August 31, 2023, from eight hospitals in the northern, central, southern, and coastal areas of China’s Zhejiang Province. This area covered 75% of the local population, including rural and urban communities as well as provincial-level tertiary, municipal, county-level, and general hospitals as well as specialized pediatric facilities (more details are available in Supplementary Figure 1 and Supplementary Table 1).

### 2.2 Study population

The participants of this present study consisted of all hospitalized children aged 5 years and younger who tested positive for either influenza A, B, or RSV, which was determined by using a combined RT-PCR or rapid antigen test of nasopharyngeal swab samples or lower respiratory tract samples during a hospital presentation during the study period. No change was found in the diagnostic criteria during the study period. Patients with a positive report for both viruses and those who had tested positive and reported in the previous month were considered to be different cases.

### 2.3 Data collection and analysis

All children aged 5 years and younger admitted to the eight hospitals during the study period were identified from the hospital information system (HIS). All RSV-confirmed inpatients were screened from the HIS, and hospitalization information was extracted from anonymized electronic medical records from the eight hospitals. Demographic variables collected for each patient encounter during the study period included age and sex; clinical characteristics included onset date, confirmed date, hospital admission and discharge dates, and presence of complex chronic conditions; outcomes assessed included discharge diagnosis, clinical outcome (s), hospital stay, and hospitalization cost (RMB). Our study only included hospitalized children younger than 5 years old who were confirmed by the eight hospitals to have RSV or influenza, we excluded outpatients with mild symptoms during the study period. There were no changes in the investigation criteria and data analyzed in different hospitals or different years.

Each patient had a unique medical record number used as an identifier to retrieve and link all variables from different databases. Records were excluded if they had data missing from the age, primary discharge, admission diagnosis, or admission date fields. Specific names, ID numbers, home addresses, and other private information were not included.

Based on 2019 national guidelines for the diagnosis and treatment of community-acquired pneumonia in children, pneumonia is defined by the presence of lung consolidation on chest imaging (radiology or computed tomography scan) interpreted by a radiologist (10). The clinical severity categories in our study included acute upper respiratory tract infections, acute bronchitis, acute bronchiolitis, and pneumonic. These four clinical diagnostic classifications are made by an attending physician during a patient’s visit to a hospital according to the clinical manifestations, imaging examinations, laboratory tests, and specific criteria of China. We extracted corresponding information from a patient’s case and conducted a statistical analysis; no modifications were made to clinical classifications.

The study population was described in terms of epidemiological characteristics and disease burden. Age groups were categorized as follows: ≤5 months, 6–12 months, 13–24 months, 25–36 months, 37–48 months, and 49–60 months. To compare the disease burden of RSV and influenza infections between the pre-pandemic and pandemic periods, the period from 1 January 2018 to 31 December 2019 was considered the pre-pandemic period, and the period from 1 January 2020 to 31 August 2023 was considered the post-pandemic period.

### 2.4 Statistical analyses

First, normality was tested for continuous variables. As the distribution of variables including age, length of hospital stay, and hospitalization cost were non normal, we calculated the median range. Categorical variables were expressed as frequencies and percentages. T-, chi-square, and Fisher’s exact tests were applied to compare continuous and categorical variables as appropriate.

Second, a multivariate logistic regression was used to estimate the odds ratio (OR) and 95% confidence interval (CI) associated with gender, age, pneumonia, severe illness, mixed infection, underlying disease, length of hospital duration, and hospitalization cost of each inpatient infected with RSV and influenza. All statistical analyses were performed using the R software program (v. 4.3.1). Statistical significance was set at *p* < 0.05 (two tails).

## 3 Results

### 3.1 Overall hospitalization burden of RSV infection vs. influenza infection

Over the five seasons between 2018 and 2023, 10,428 hospitalized children aged ≤5 years (7,857 RSV infections and 2,571 influenza infections) were enrolled in this study. RSV was responsible for approximately three times as many hospitalizations as influenza in the ≤5 years age group (1,571 vs. 514 cases per year) (Table 1). The hospitalization rate of RSV cases was higher than that of influenza cases in the same year (Supplementary Table 2).

**Table 1:** Comparison of the disease burden of hospitalization attributable to influenza and RSV among children 0–5 years old during 2018–2023 in Zhejiang Province, China.

Variable	RSV	Influenza	Test value^a^	*p* value^a^
Overall No. (%)	7,857 (75.35)	2,571 (24.65)		
Stages No. (%)				
Prepandemic years	1,081 (13.75)	622 (24.19)	154.36	< 0.001
Pandemic years	6,776 (86.25)	1,949 (75.81)		
Gender No. (%)				
Male	4,746 (60.40)	1,520 (59.12)	1.331	0.249
Female	3,111 (39.60)	1,051 (40.88)		
Age (Months) (median, range)	12 (0–60)	36 (0–60)	−37.29	< 0.001
No. (%)				
0–5	2,374 (30.22)	336 (13.01)	1,798.47	< 0.001
6–12	2,806 (35.71)	440 (17.11)		
13–24	1,133 (14.42)	367 (14.27)		
25–36	1,049 (13.35)	530 (20.61)		
37–48	376 (4.79)	451 (17.54)		
49–60	119 (1.51)	447 (17.39)		
Underling diseases No. (%)				
Yes	1,417 (18.03)	374 (14.55)	16.57	< 0.001
No	6,440 (81.97)	2,197 (85.45)		
Mixed infections No. (%)				
Yes	1,601 (20.38)	367 (14.27)	47.08	< 0.001
No	6,257 (79.62)	2,204 (85.73)		
Clinical severity No. (%)				
Pneumonia	6,360 (80.95)	1,032 (40.14)	3,421.03	< 0.001
Acute bronchitis	754 (9.60)	532 (20.69)		
Acute bronchiolitis	652 (8.29)	19 (0.74)		
Acute upper respiratory tract infection	91 (1.16)	988 (38.43)		
Clinical outcome	All cases were discharged	All cases were discharged		
Hospitalization duration(days) (median, range) (ratio)	5.00 (1–64)	4.00 (1–62)	8.30	< 0.001
Underling diseases(yes/no)	1.20 (6.00/5.00)	1.25 (5.00/4.00)	/	/
Mixed infections(yes/no)	1.00 (5.00/5.00)	1.25 (5.00/4.00)	/	/
Pneumonia(yes/no)	1.00 (5.00/5.00)	1.25 (5.00/4.00)	/	/
Hospitalization expenses (RMB) (median, IQR; Range) (ratio)	Median: 4460.73 (IQR: 3,364.98–6207.55; Range: 684.29–285,655.90)	Median: 3664.00 (IQR: 2,732.41–5,016.66; Range: 340.00–352,008.68)	6.11	0.023
Underling diseases (yes/no)	1.49 (6244.62/4,197.73)	1.53 (5,302.28/3,456.85)	/	/
Mixed infections (yes/no)	1.07 (4,704.31/4,395.61)	1.49 (5226.10/3,497.51)	/	/
Pneumonia (yes/no)	1.13 (4,558.51/4,047.55)	1.40 (4,524.24/3,227.82)	/	/

RSV = respiratory syncytial virus; RMB = Renminbi; IQR = interquartile range.

^ap-value is used to assess the difference between the RSV group and influenza group in the corresponding classification. The test value of count data is the chi-square test, and the test value of quantitative data is the T-test.^

For population distribution, there was no difference between males and females in the two groups (*p* = 0.249). However, the mean age was lower for RSV than for influenza (15.92 vs. 31.75 months; *p* < 0.001); the 0–2 years group accounted for approximately 80% of RSV infections, and the 3–5 years group accounted for 55.54% of influenza virus infections (Table 1).

**Figure 1:**
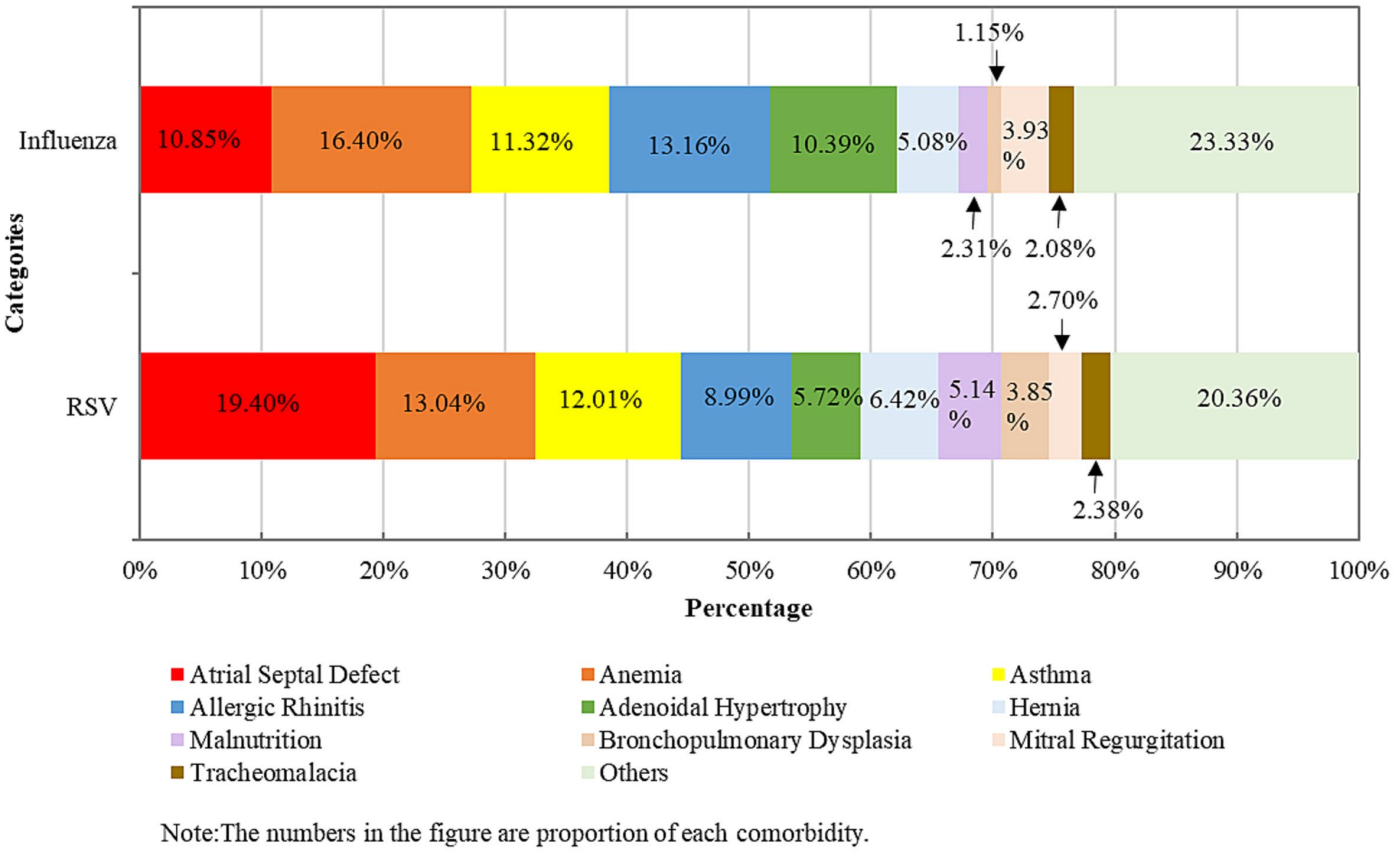
Comparison of the underlying diseases of hospitalization due to respiratory syncytial virus and influenza infections among children ≤5 years old in 2018–2023 in Zhejiang Province, China.

Regarding comorbidities, 18.03% of RSV inpatients and 14.55% of influenza inpatients had at least one underlying medical condition, respectively (*p* < 0.001) (Table 1). The categories of comorbidities of RSV and influenza inpatients were similar; the top five most common comorbidities in the RSV groups were atrial septal defect (21.32%), anemia (14.33%), asthma (13.20%), allergic rhinitis (9.88%), and adenoidal hypertrophy (6.28%); for influenza inpatients, the most common comorbidities were anemia (18.99%), allergic rhinitis (15.24%), asthma (13.11%), atrial septal defect (12.57%), and adenoidal hypertrophy (12.04%) (Figure 1; Supplementary Table 3).

For coinfections, the percentage in the RSV groups was greatly higher than that in the influenza groups (20.38% vs. 14.27%; *p* < 0.001). The categories of pathogens resulting in mixed infections in RSV and influenza hospitalized patients were similar. In terms of viral coinfection, the top three most common viruses were human rhinovirus, cytomegalovirus, and human adenovirus in the RSV groups, and human rhinovirus, rotavirus, and human adenovirus in the influenza groups (Supplementary Figure 2A). The top three most common bacteria for both the RSV and influenza groups were mycoplasma pneumonia, streptococcus pneumonia, and haemophilus influenza (Supplementary Figure 2B).

For disease burden, infections in the RSV groups, with a higher percentage of pneumonia, were more severe than those in the influenza groups (80.95% vs. 40.14%; *p* < 0.001). Inpatients with RSV had longer hospitalization duration (median: 5.00 vs. 4.00 days; *p* < 0.001) and hospitalization cost (median: 4,460.73 vs. 3,664.00 RMB; *p* = 0.023) than those with influenza (Table 1). The distributions of hospital stays and hospitalization costs are shown in Supplementary Figure 3.

### 3.2 Epidemiological changes during the pre- and post-pandemic periods

The number of hospitalizations due to RSV and influenza was higher in the post-pandemic period than in the prepandemic period (6,776 vs. 1,081 cases for RSV; 1,949 vs. 622 cases for influenza, respectively; *p* < 0.001) (Table 1).

**Figure 2:**
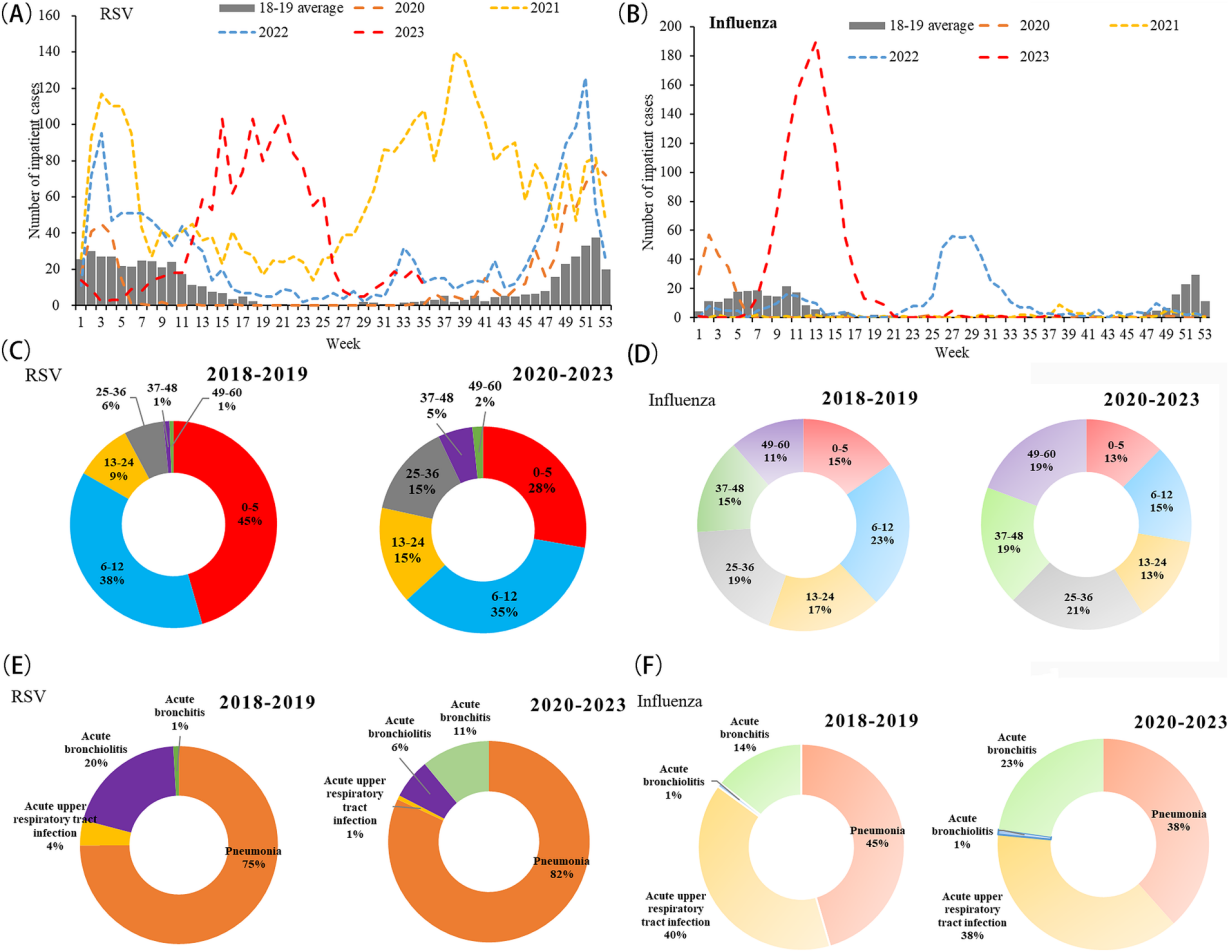
Hospitalization trends, age distribution, and disease spectrum in hospitalized children younger than 5 years with respiratory syncytial virus and influenza infections before and after the COVID-19 pandemic. **(A)** Temporal distribution of RSV (number of cases: 1,081 in 2018–2019 and 6,776 in 2020–2023); **(B)** temporal distribution for influenza (number of cases: 622 in 2018–2019 and 1,949 in 2020–2023); **(C)** age distribution for RSV; **(D)** age distribution for influenza; **(E)** disease spectrum for RSV; **(F)** disease spectrum for influenza.

In 2018–2019, RSV and influenza epidemics typically followed seasonal patterns, peaking in December or January, but the COVID-19 pandemic disrupted RSV seasonality in 2020–2023 (Figure 2). In 2020–21, the typical winter RSV and influenza epidemics did not occur. RSV circulation began earlier and continued longer in 2021–2022 than during the prepandemic years. The 2022–2023 season started later than the 2021 season but earlier than the prepandemic seasons. The peak of the RSV season was delayed to the spring–summer of 2023 (Figure 2A). In contrast, influenza showed an off-season peak in July 2022 and March 2023 (Figure 2B).

A shift toward older children with RSV symptomatic infections was observed in the post-pandemic period. For the RSV groups, the median age was larger for pandemic years than for pre-pandemic years (16.86 vs. 10.09 months; χ^2^ = 217.62, *p* < 0.001). This increase was primarily observed among children aged 13–24 months, representing 9% of all RSV hospitalizations from 2018 to 2019 to 15% in 2020–2023. In contrast, compared with pre-pandemic seasons, the proportion of the ≤5 months group (45% vs. 28%) and the 6–12 month group (38% vs. 35%) decreased in the pandemic seasons for RSV infection cases (Figure 2C). Influenza groups showed a similar trend; the median age was 27.42 months in 2018–2019 to 33.13 months in 2020–2023 (χ^2^ = 45.66, *p* < 0.001) (Figure 2D). The most noticeable increase was observed in the 37–48 month group (15% vs. 19%) and the 49–60 month group (11% vs. 19%), respectively.

Disease severity parameters showed an increase in RSV groups during pre-pandemic seasons vs. pandemic seasons (percentage of acute bronchiolitis: 1% vs. 11%) (Figure 2E). For influenza infection, this percentage of influenza increased from 14 to 23% in 2020–2023 compared with 2018–2019 (Figure 2F).

### 3.3 Disease burden during the pre- and post-pandemic periods

**Figure 3:**
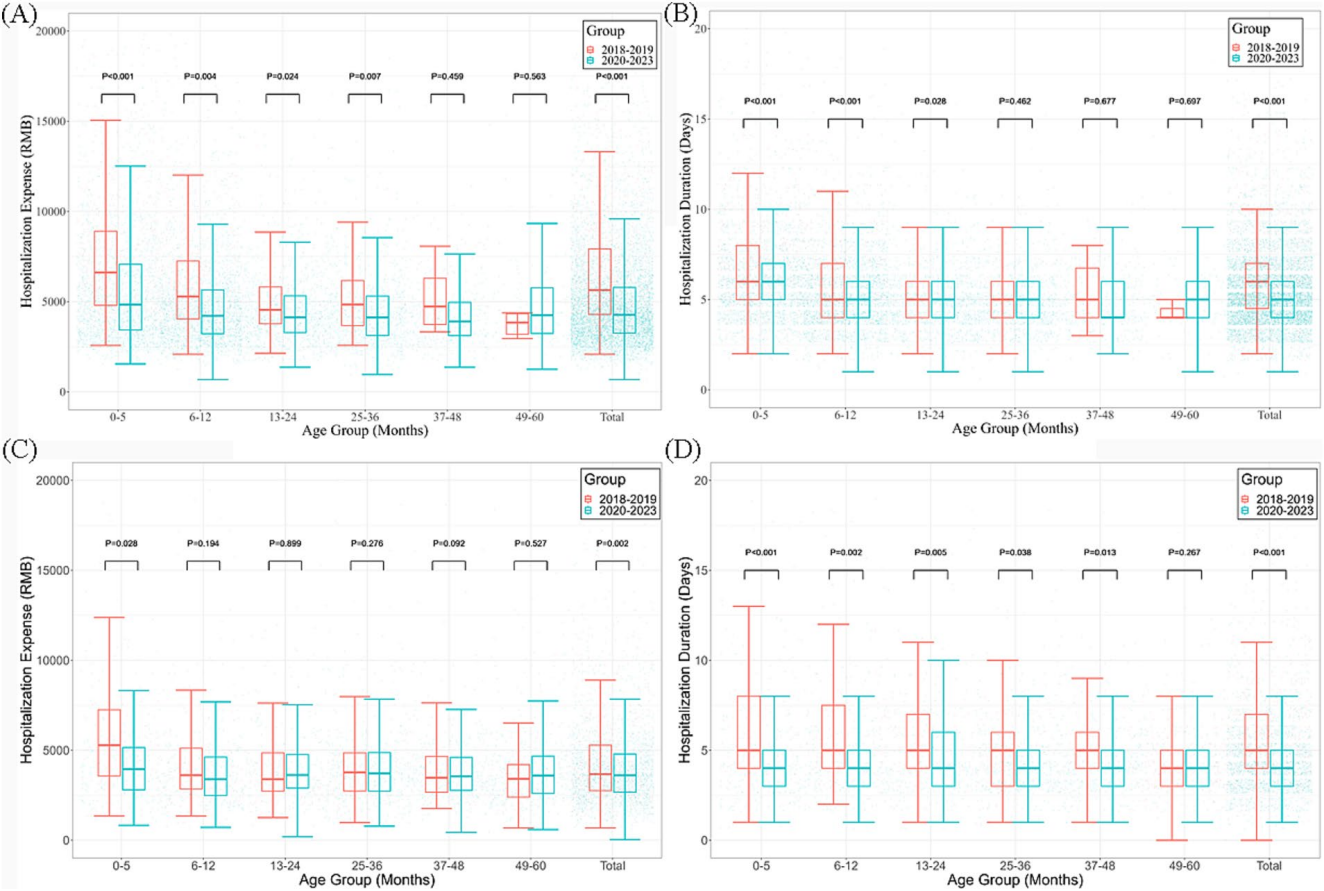
Median hospital stay and median hospitalization cost of inpatients due to influenza and RSV infections among children ≤5 years old before and after the COVID-19 pandemic in Zhejiang Province. **(A,B)** Hospitalization costs and hospital stays for RSV infection, respectively; **(C,D)** Hospitalization costs and hospital stays for influenza infections, respectively.

The average hospitalization cost (4299.29 vs. 5697.51 RMB; *p* < 0.001) and duration (5 vs. 6 days; *p* < 0.001) for RSV inpatients in 2020–2023 were significantly lower than those in 2018–2019. Analyses stratified by age showed that disease burden significantly decreased among the groups aged younger than 6 months and 6–12 months separately (all *p* < 0.05) (Figures 3A,B).

The hospitalization cost (3,631.03 vs. 3,742.585 RMB) and duration (4 vs. 5 days) for influenza inpatients in 2020–2023 were also significantly lower than those in 2018–2019 (*p* < 0.001). Analyses stratified by age showed a downward trend for disease burden among the groups aged younger than 2 years (all *p* < 0.05) (Figures 3C,D).

### 3.4 Risk factors for hospitalization burden

**Figure 4:**
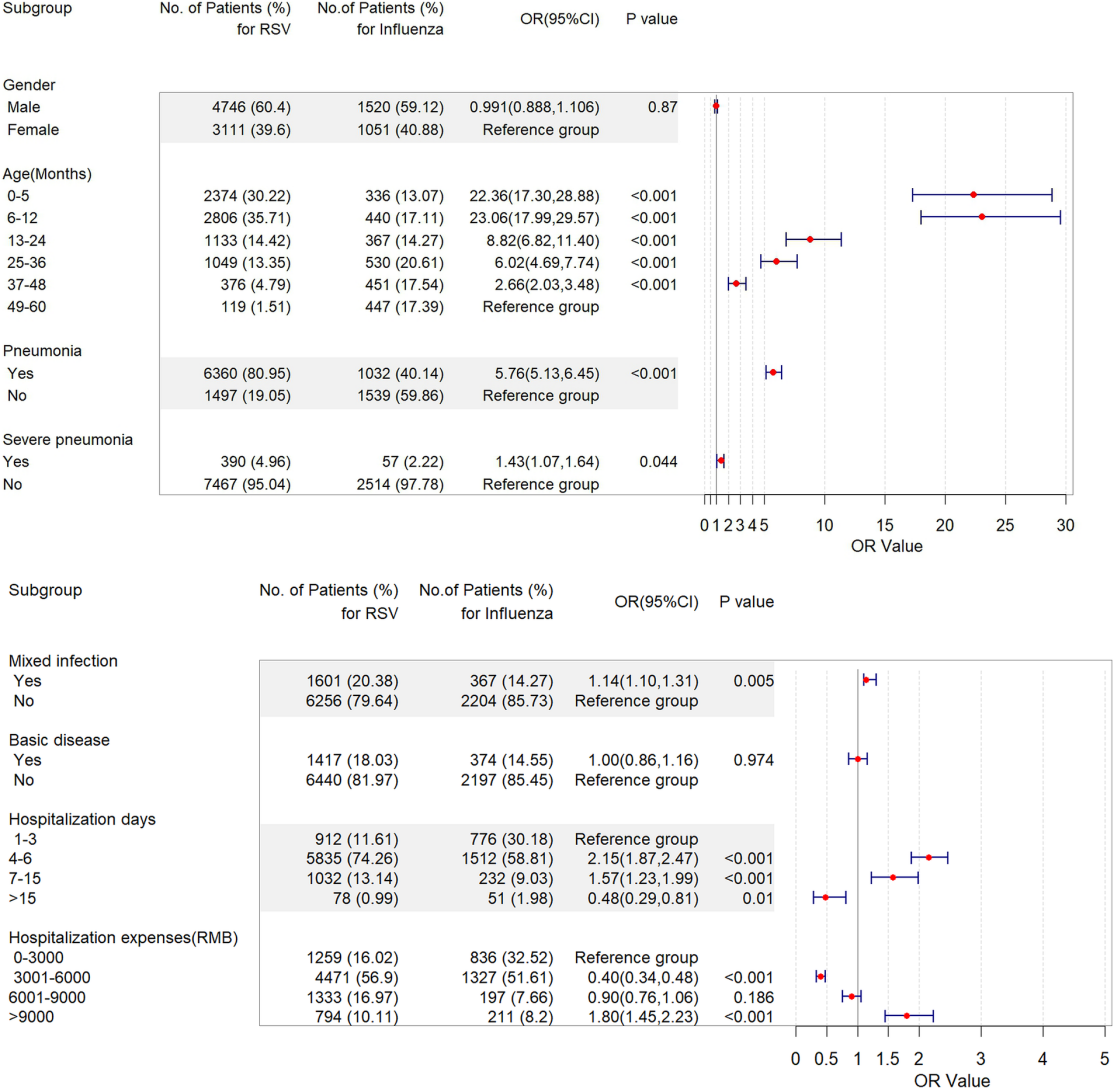
Forest plot of a multivariate linear regression model for the predictors of children aged ≤5 years hospitalized with respiratory syncytial virus or influenza infection in 2018–2023 in Zhejiang Province. CI, confidence interval; OR, odds ratio; RSV, respiratory syncytial virus.

A multivariate logistic regression analysis revealed that the odds of all variables were significantly higher among patients hospitalized with RSV compared with those hospitalized with influenza (Figure 4). This analysis confirmed that, compared with influenza, RSV was related to an increased burden in all groups; the greatest burden was seen in the 6–12 months group (OR = 23.1; 95% CI, 18.0–29.6), followed by the ≤5 months group (OR = 22.4; 95% CI, 17.3–28.9), 13–24 months group (OR = 8.8; 95% CI, 6.80–11.4), 25–36 months group (OR = 6.0; 95% CI, 4.7–7.7) and 37–48 months group (OR = 2.7; 95% CI, 2.0–3.5) (Figure 4). Compared with influenza patients, RSV patients had a higher risk of pneumonia (OR = 5.8; 95% CI, 5.1–6.5), severe pneumonia (OR = 1.4; 95% CI, 1.15–1.6), and mixed infection (OR = 1.1; 95% CI, 1.1–1.3) (Figure 4).

Compared to the influenza groups, the RSV group had a higher risk for the average length of hospital stay [4–6 days for (OR = 2.1; 95% CI, 1.9–2.5); 7–15 days (OR = 1.6; 95% CI, 1.2–2.0)]. However, the risk of having to be hospitalized for 15 days or more was lower in the RSV groups (OR = 0.5; 95% CI, 0.3–0.8). On the same, RSV patients had higher hospitalization costs [>9,000 RMB, OR = 1.80; 95% CI, 1.5–2.2], and lower costs in the group [3,001–6,000 RMB, OR = 0.4; 95% CI, 0.3–0.5] compared with influenza patients (Figure 4).

## 4 Discussion

In this retrospective observational study from multiple centers in Zhejiang Province, 7,857 RSV cases and 2,571 influenza cases were analyzed for nearly five epidemic seasons of RSV and influenza between 2018 and 2023. This study found that RSV was associated with a higher risk of disease burden (younger age, higher percentage of comorbidities, coinfections, pneumonia, hospitalization costs, prolonged hospital stays) compared with influenza in children aged 5 years and younger. NPIs during the COVID-19 pandemic had varied impacts on both RSV and influenza infections. Compared with the pre-pandemic period, the number of cases, ages of inpatients, and percentage of pneumonia significantly increased during the pandemic years, whereas hospitalization cost and hospital stay per admission decreased slightly.

Previous studies showed that children under 5 years old were the high-risk group for RSV infection. Cui et al. found that children under 5 years old accounted for 87.13% of all RSV and 8.34% of influenza-infected patients in nine provinces of China (19). In our study, compared with influenza, RSV was associated with higher health care costs, leading to hospital overcrowding for all age groups, with the highest risk observed in children aged 12–24 months. These findings align with recent studies (20). For example, in the UK, RSV-attributable disease leads to 20,000–30,000 hospitalizations annually, which is significantly higher than influenza (up to 20 times higher for children under 6 months) and accounts for over 55,000 annual bed days (21).

These results may be explained by several reasons. First, RSV has a much higher proportion of pneumonia cases compared to influenza, whereas influenza has a higher proportion of acute upper respiratory tract infections. Therefore, pneumonia (OR = 5.8) and severe pneumonia (OR = 1.4) were more frequent in the RSV group than in the influenza group. Furthermore, RSV infections are more frequently associated with coinfections (22, 23). In Singapore, for RSV infection in children less than 6 months of age, the average cost per bronchiolitis hospitalization was US$2,209, rising to US$5,942 for hospitalization for pneumonia with complications (22).

Second, this study also confirmed that hospitalized patients with RSV were younger than those with influenza (16 vs. 32 months); the findings are consistent with two previous studies examining RSV age (24, 25). Younger patients presented with higher respiratory signs and symptoms (dyspnea, oxygen desaturation, and wheezing), characteristic of the pathophysiology of RSV infection (26). Third, Oseltamivir is a specific antiviral drug that is effective in reducing the disease burden of influenza (27).

Compared with other countries, the mean length of hospital stay in this current study was longer among children aged ≤5 years (5.66 days in RSV and 5.06 days in influenza) than RSV infections reported in other countries. For example, one study reported that across Europe, the average length of hospital stay for RSV infection was 2–4 days (28). In Brazil, the median length of hospital stay for influenza was 3.0 days, while in Mexico, the median length of hospital stay was 5.0 days (29).

Conversely, the median direct medical cost for hospitalization of children with RSV (5,616.12 RMB) and influenza-related (5,352.99 RMB) illnesses was lower in China than in other countries. The average cost of RSV-associated hospitalization was estimated at US $3,300 in Japan and at US$4,500 in Australia (22). The cost of influenza hospitalization in the USA was $3,366–$19,444 (30). Compared with other domestic research results, the average cost of RSV-related hospitalization cases was lower than the research results of Ren et al. on the direct economic burden caused by RSV infection in hospitalized children aged 0–59 months in Henan Province (1055.3 US dollars (US$) (95% CI: 998.2–1112.5 US$) for each episode) (31). This difference might be due to the different levels of economic development and medical care systems.

The COVID-19 pandemic had a notable impact on the circulation of seasonal respiratory viruses, including RSV and influenza virus (32). For RSV groups, the usual winter peak did not occur in the winter of 2020, which was like studies from the USA and UK, when most COVID-19 restriction measures stopped, RSV infections and hospital admissions rose sharply in the summer of 2021, autumn of 2022, and spring of 2023 (33). However, compared with 2018–2019, the total number of RSV hospitalization cases in 2020–2023 remained high in all age groups of patients ≤5 years, suggesting higher intensity of circulation (32). Similarly, in 2020–2021, the intensity of influenza activity continued to decrease significantly, while it increased in the summer of 2022 and the spring of 2023.

This result is consistent with the research results of Cui AL et al. on the epidemiological characteristics of RSV infection in 16 provinces of China (excluding Zhejiang Province) from January 2009 to September 2023 (34) and the study by Li MZ et al. on RSV epidemiology in Beijing during 2015–2023 (35). This altered activity patterns between the two viruses hinted that the public health measures against COVID-19 had a more effective impact on influenza than RSV (34). One plausible reason for this observation could be the ability of RSV to survive for a longer duration out of a host and to spread quickly by direct contact (36). Also, children, particularly those under 1 year, could not fully benefit from preventive measures during the pandemic of COVID-19, such as wearing masks.

In our study, compared with the clinical severity of RSV cases in 2017–2018, our results showed a significant increase in the percentage of acute bronchiolitis for RSV and influenza. We propose three hypotheses for this phenomenon. First, during the COVID-19 pandemic, PCR techniques were widely used in hospitals, resulting in the timely, accurate detection of specific or broad-spectrum pathogens (10). This initiated specific treatments and reduced the occurrence of complications and disease burden (37). Second, we found that during pandemic seasons, the age distribution of children infected with RSV or influenza decreased with the proportion of children aged 0–12 months decreasing and the average age increased. A similar older age structure of RSV and influenza patients was observed in the USA, France, and Australia; this situation could be attributed to ‘immunity debt’ resulting from the accumulation of immune-naive children due to the strict implementation of NPIs in 2020–2023 (33). This older age population influenced a host’s immune response and thus increased its ability to fight against both viruses and reduce the disease burden. Third, more of the population appeared to be vaccinated against influenza during the pandemic years, demonstrating a reduction in hospitalization burden. Consistent with the above results, the disease burden (including expenses and duration) of each RSV or influenza-infected patient was lower during the pandemic period than during the pre-pandemic period.

In this retrospective cross-sectional study, we report a surge in RSV and influenza infections in older children from 2020–2023. Meanwhile, we found that the disease burden of children infected with RSV and influenza was severe. Increased cases and disease burdens may continue to pose considerable strains on healthcare systems. In response to these challenges, clinicians and program planners should take measures to increase China’s health care capacity and ICU (intensive care unit) capacity to meet greater demands for RSV and influenza admissions. Policymakers have introduced imminent new RSV and influenza prevention strategies, including increased overall influenza vaccine coverage and approved RSV monoclonal antibodies in China, to minimize the burden of the two diseases in this age group.

The advantage of this study is the large sample, multicenter survey, and comparative study of the epidemiology characteristics and disease burden of RSV and influenza infections. However, this study has several limitations due to differences in the criteria for admission, treatment plans, and laboratory testing capabilities in several hospitals. Additionally, the lack of relevant data on viral genotyping, family economic status, and indirect economic burden may influence whether parents decide to hospitalize their children, which is also useful in evaluating epidemiological features and disease burdens.

## 5 Conclusion

This study, conducted at eight hospitals for nearly five consecutive epidemic seasons of RSV and influenza, included the largest cohort of RSV- and influenza-infected hospitalized children under 5 years of age. This study indicates that the disease burden of RSV in all groups aged ≤5 years, especially those under 1 year, was significantly higher than that of influenza; however, the burden of RSV and influenza infections per admission was reduced during the pandemic compared with the prepandemic period. The seasonal pattern of the two viruses was significantly disrupted by the easing of COVID-19 restriction measures, but RSV infections remained at a higher level than influenza infections during the pandemic (38). It is uncertain whether this change will continue in upcoming seasons.

Future studies should focus on specific mechanisms via the seasonality and epidemiologic characteristics of RSV and influenza. Second, it is critical to conduct a comprehensive study on RSV and influenza immunity at the population level and to inform the prioritization of immunization platforms. Third, the safety of RSV and influenza vaccines as well as monoclonal antibody immunogenicity should be thoroughly assessed.

## Data Availability

The raw data supporting the conclusions of this article will be made available by the authors, without undue reservation.
