# Supplementary material for: The epidemiology and burden of respiratory syncytial virus and influenza infections in hospitalized children under 5 years old in Zhejiang, China (2018–2023)
Source: Front Public Health. 2025 Mar 19;13:1470770. doi: 10.3389/fpubh.2025.1470770 (PMC11966739; doi:10.3389/fpubh.2025.1470770)
Supplement: Supplementary file 1 [file Data_Sheet_1.docx]

**Supplementary file**

**Supplementary Figure 1.** The geographical location and characteristics of hospitals in this study

Information of geographical location and characteristics of eight hospitals


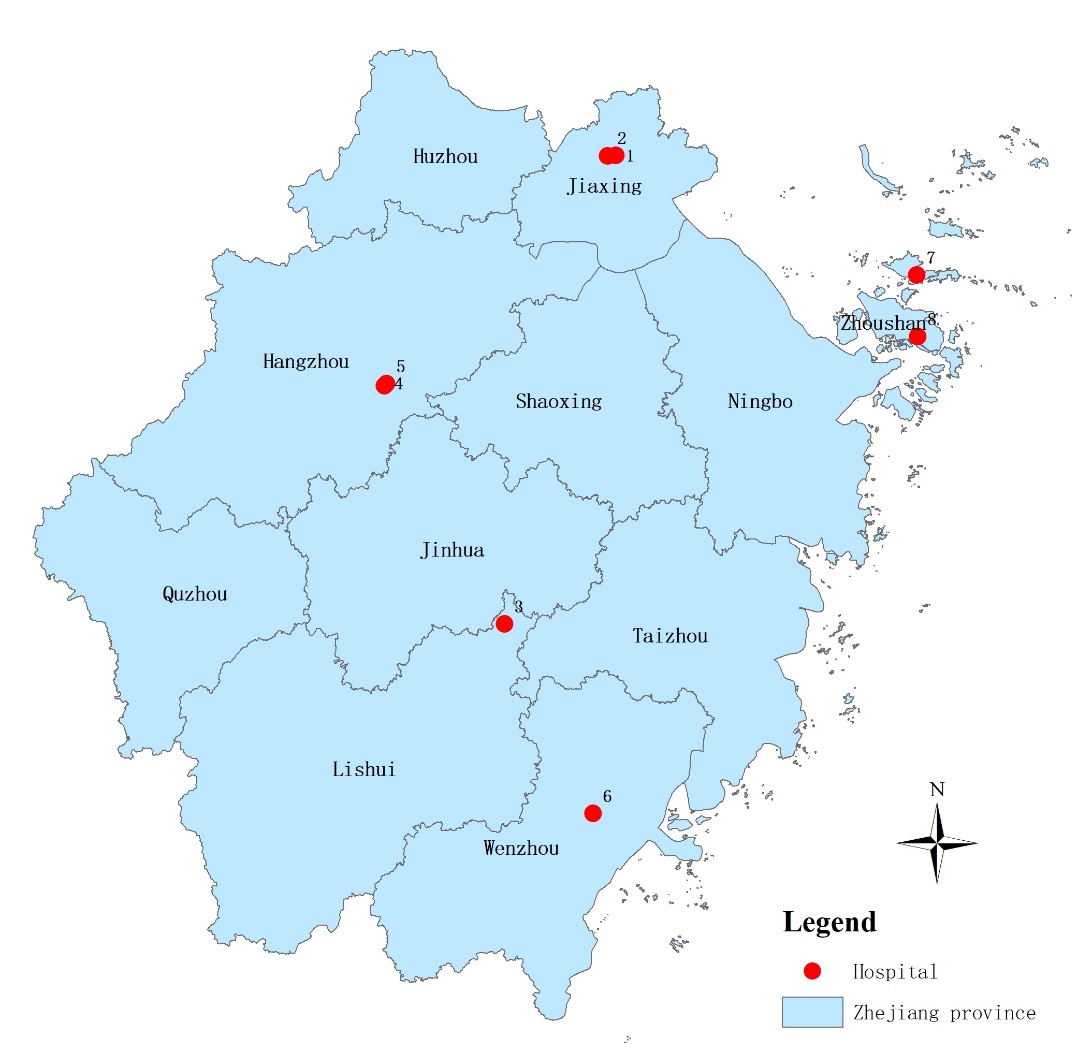


**Supplementary Table 1 Characteristics of hospitals in this study**

| **Number** | **Hospital** | **Grade** | **Type** |
| --- | --- | --- | --- |
| 1 | Jiaxing Maternity and Child Health Care Hospital | Level III Grade A | Women and Children |
| 2 | The First Hospital of Jiaxing | Level III Grade A | Comprehensive hospital |
| 3 | The Second People's Hospital of Jinyun | Level II Grade B | Comprehensive hospital |
| 4 | The Maternity and Child Health Hospital of Tonglu | Level II Grade B | Comprehensive hospital |
| 5 | The First People's Hospital of Tonglu | Level II Grade A | Comprehensive hospital |
| 6 | Wenzhou Medical College Affiliated Second Hospital Wenzhou Medical College Affiliated Yuying Children's Hospital | Level III Grade A | Women and Children |
| 7 | People's Hospital of Daishan | Level II Grade A | Comprehensive hospital |
| 8 | Zhoushan Hospital | Level III Grade A | Comprehensive hospital |

Supplementary Table 2 Annual hospitalization rate of RSV and influenza.

| Year | No. of RSV hospitalization (hospitalization ratio, %) | No. of Influenza  hospitalization (hospitalization ratio, %) | Total number of hospitalizations |
| --- | --- | --- | --- |
| 2018 | 518(1.07) | 147(0.30) | 48435 |
| 2019 | 563(1.19) | 475(1.00) | 47407 |
| 2020 | 637（1.96） | 194(0.60) | 32437 |
| 2021 | 3344(6.93) | 60(0.12) | 48246 |
| 2022 | 1505(3.42) | 533(1.21) | 43983 |
| 2023 | 1285(4.00) | 1162(3.61) | 32150 |
| Total | 7852(3.11) | 2571(1.02) | 252658 |

Supplementary Table 3 Comparison of the underlying diseases of hospitalisation due to respiratory syncytial virus and influenza infections among children ≤5 years old in 2018–2023 in Zhejiang Province, China.

| Underlying diseases | **No. of RSV** | **No. of Influenza** | **Total** |
| --- | --- | --- | --- |
| Atrial Septal Defect | 302 | 47 | 349 |
| Anemia | 203 | 71 | 274 |
| Asthma | 187 | 49 | 236 |
| Allergic Rhinitis | 140 | 57 | 197 |
| Adenoidal Hypertrophy | 89 | 45 | 134 |
| Hernia | 100 | 22 | 122 |
| Malnutrition | 80 | 10 | 90 |
| Bronchopulmonary Dysplasia | 60 | 5 | 65 |
| Mitral Regurgitation | 42 | 17 | 59 |
| Tracheomalacia | 37 | 9 | 46 |
| Ventricular Septal Defect | 37 | 8 | 45 |
| Epilepsy | 22 | 21 | 43 |
| Patent Ductus Arteriosus | 37 | 5 | 42 |
| Congenital Heart Disease | 26 | 8 | 34 |
| Hypothyroidism | 24 | 5 | 29 |
| Congenital Laryngeal Chondromalacia | 21 | 0 | 21 |
| Tracheal Stenosis | 14 | 6 | 20 |
| Cerebral Hypoplasia | 12 | 6 | 18 |
| Premature Birth | 17 | 0 | 17 |
| Congenital Laryngeal Stridor | 13 | 3 | 16 |
| Congenital Bronchial Malformation | 14 | 1 | 15 |
| Gallstone | 10 | 4 | 14 |
| Vitamin D Deficiency | 5 | 8 | 13 |
| Pulmonary Hypertension | 9 | 3 | 12 |
| Hyperuricemia | 4 | 6 | 10 |
| Cholestasis | 8 | 1 | 9 |
| Cardiac Insufficiency | 8 | 0 | 8 |
| Reflux | 8 | 0 | 8 |
| Accessory Spleen | 6 | 2 | 8 |
| Obesity | 6 | 1 | 7 |
| Kidney Stone | 6 | 0 | 6 |
| Kawasaki Disease | 4 | 2 | 6 |
| Glucose/6/Phosphatase Deficiency | 4 | 2 | 6 |
| Leukemia | 0 | 5 | 5 |
| Immunodeficiency | 2 | 2 | 4 |
| Cerebral Palsy | 0 | 2 | 2 |

**Supplementary Figure 2.** Pathogen distribution of viral and bacterial co-infections in hospitalisation due to respiratory syncytial virus and influenza infections among children ≤5 years old in 2018–2023 in Zhejiang Province, China.

Notes: a: viral co-infections; b: bacterial co-infections.


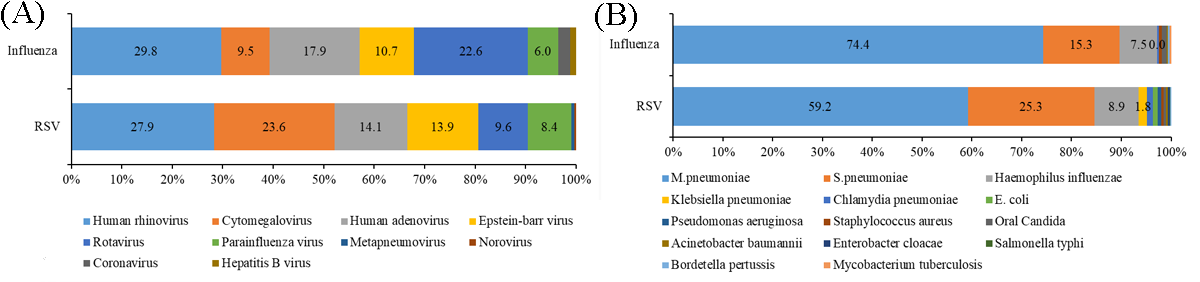


**Supplementary Figure 3. The distribution of hospital stays and hospitalization costs of inpatient attributable to influenza and RSV among children 0–5 years old between 2018 to 2023, in Zhejiang Province, China**


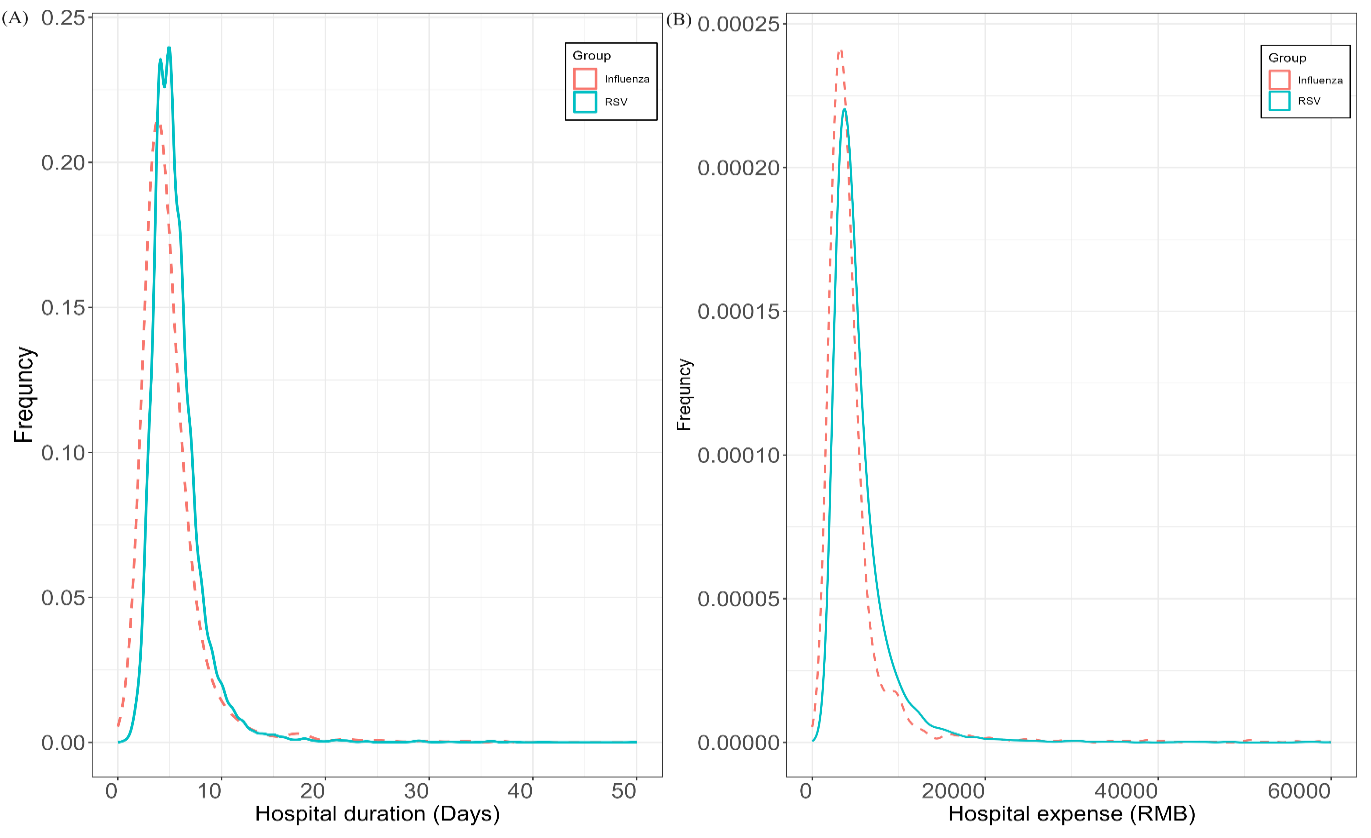


**Supplementary material:**

**Relevant definition:**

1. Influenza-like illness (ILI) is a non-specific syndrome defined as fever (temperature of 38°C or greater) with cough or sore throat (National Guideline for Institutional Outbreak Management of Seasonal Influenza, 2018 version).

2. Severe acute respiratory infection (SARI) is defined as an acute respiratory infection with symptoms including cough and fever within 10 days of presentation and with hospitalization (The World Health Organization [WHO] Global Influenza Programme, https://www.who.int/teams/global-influenza-programme/surveillance-and-monitoring/case-definitions-for-ili-and-sari).
